# Endocrine effects of MEK and BRAF inhibitor therapy in paediatric patients: a tertiary centre experience

**DOI:** 10.1007/s11060-024-04896-9

**Published:** 2024-12-13

**Authors:** Arif Hanafi Bin Jalal, Harriet Gunn, Buddhi Gunasekara, Hoong-Wei Gan

**Affiliations:** 1 UCL Medical School, University College London, London, UK; 2 Department of Endocrinology, University College London Hospitals NHS Foundation Trust, London, UK; 3 UCL Great Ormond Street Institute of Child Health, University College London, London, UK; 4 Present Address: Department of Endocrinology, Great Ormond Street Hospital for Children NHS Foundation Trust, London, UK

**Keywords:** Hyponatraemia, MEK inhibitor, BRAF inhibitor, Paediatric oncology, Paediatric endocrinology, MAPK/ERK pathway

## Abstract

**Purpose:**

BRAF and MEK inhibitors are used to treat a range of paediatric tumours including low-grade gliomas. The ubiquitous nature of the BRAF/MAPK/MEK pathway means such treatments are not without side effects such as renal tubulopathies and hyperglycaemia. This study aims to describe the endocrine dysfunction observed in a cohort of children treated with BRAF and MEK inhibitors at the largest paediatric centre in the UK utilising these treatments.

**Methods:**

Electronic data for patients treated with dabrafenib (BRAF inhibitor) and trametinib (MEK inhibitor) from January 2019 to May 2022 were retrospectively reviewed. Outcomes included diagnosis of glucose dysregulation, the presence of hyponatraemia (< 135 mmol/l) and sodium nadir during treatment.

**Results:**

A total of 55 patients were included for analysis. Nine patients had at least one hyponatraemic episode during treatment of whom three had coexisting central diabetes insipidus. A statistically significant difference (p-value = 0.037) with regards to the plasma sodium nadir during treatment was observed between patients with diabetes insipidus (median = 134 (132–137) mmol/l) and patients without (median = 137 (127–141 mmol/l). Six patients were diagnosed with a form of glucose dysregulation (e.g. insulin resistance, type 2 diabetes), of whom four were diagnosed during treatment with dabrafenib, all with hypothalamo-pituitary lesions.

**Conclusion:**

Clinicians using such treatments need to be aware of these potential effects, particularly the risk of hyponatraemia in patients with pre-existing central diabetes insipidus and monitor for these accordingly, including performing measurements of sodium and glucose prior to, during and after treatment.

## Introduction

The MAPK/ERK signalling pathway has been shown to play a role in the development of various benign and malignant tumours, being involved in both tumour suppression and oncogenesis. Mutations in downstream kinases such as BRAF and MEK have been demonstrated to be suitable targets for molecular therapy, potentially reducing off-target effects associated with traditional methods of oncological treatments such as chemotherapy and radiotherapy [1]. The efficacy of BRAF and MEK inhibition has been demonstrated in adult populations for melanoma and more recently in non-small cell lung cancer [2, 3].

Central nervous system tumours are the second commonest cancer in childhood and adolescence, accounting for 25% of cancer cases in children aged 0–14 in the United Kingdom, with low-grade gliomas occurring most frequently [4]. Complete surgical resection is the treatment of choice, but when these tumours are located in eloquent areas of the brain such as the suprasellar compartment, this is rarely accomplished, therefore requiring radiotherapy and chemotherapy. However, such treatments are not without their own disadvantages, with radiotherapy being associated with cognitive impairment [5, 6], and chemotherapy leading to various toxicities including neuropathies, hearing impairment and infertility.

The BRAF V600E mutation, a target for current molecular therapies, has previously been detected in various childhood tumours such as pleomorphic astrocytomas (66.7%), Langerhans cell histiocytosis (LCH) (57%) and low-grade gliomas [7–9]. Tumours with BRAF V600E mutations have also shown greater susceptibility to MEK inhibition compared to wild-type BRAF cells, providing a rationale for combination therapy [10]. Dabrafenib (BRAF inhibitor) and Selumetinib (MEK inhibitor) have already demonstrated both tolerability and clinical activity in paediatric patients with solid tumours such as gliomas, neuroblastomas and thyroid cancers during phase I and II trials [11–13]. Similarly, ongoing studies in MEK inhibitors such as trametinib have shown it is well tolerated in the treatment of gliomas and plexiform neurofibromas [14, 15]. However, long-term studies are still required to evaluate their long-term safety profile in comparison with previous mainstays of treatment such as radiotherapy and chemotherapy.

The ubiquitous nature of this pathway in various physiological processes means that these treatments are not without their own side effects. Hyponatraemia (of grade III-IV severity according to the National Cancer Institute Common Terminology Criteria for Adverse Events) has been found to occur in up to 8% of patients treated with both BRAF and MEK inhibitors for non-small cell lung cancer with the risk of hyponatraemia increasing with dual inhibition treatment [16]. Hyperglycaemia has also been associated with BRAF and MEK inhibition, with the incidence of grade III-IV hyperglycaemia ranging from 2 to 6% in melanoma and 46% in thyroid cancer [17, 18]. Though currently thought to be significant only in diabetic patients; in paediatric patients this is of particular concern as the indications for such treatment include suprasellar lesions such as low-grade gliomas and LCH which already predispose patients to hypothalamo-pituitary dysfunction including hypothalamic obesity, insulin resistance and type 2 diabetes [19].

The current literature reporting endocrine toxicities associated with MAPK/ERK inhibition in children has been limited to single case reports and case series, particularly with trametinib in the presence and absence of underlying endocrine dysfunction [20, 21]. Larger cohorts are needed to better characterise the endocrine side effects observed. In this study, we describe the endocrine dysfunction observed in paediatric patients on MEK and BRAF inhibitors with regards to sodium and glucose homeostasis in the largest paediatric centre in the United Kingdom using these therapies.

## Patients and methods

### Study design and participants

Great Ormond Street Hospital (GOSH) is a tertiary paediatric centre and the largest in the UK utilising MEK and BRAF inhibitors to treat a range of paediatric conditions. We undertook a retrospective case review of electronic records for patients treated with dabrafenib and/or trametinib between January 2019 and May 2022. Patients starting therapy before this date were also included provided they were on the drug(s) during the aforementioned date range. Patients starting therapy outside our centre were excluded.

### Outcomes

Outcome data were collected at three time points: prior to the start of treatment (of at least one drug), during treatment and after treatment. For data prior to treatment the values recorded closest to the date of starting treatment were used. For data relating to post-treatment outcomes, values recorded closest to the date of data collection (May 2022) were used. Outcomes included patient weight, BMI and Z-score according to growth chart measurements, random blood glucose, insulin and HbA1c concentrations, and patient hyponatraemia (defined as readings < 135 mmol/L). A binary outcome (hyponatraemia present or not present) was used for patients before and after treatment. Additionally, the plasma sodium nadir recorded whilst patients were on treatment was also included.

Impaired glucose tolerance was diagnosed using standard WHO criteria with 2-hour oral glucose tolerance test (OGTT) blood glucose concentrations of ≥ 7.8 and < 11.1mmol/l, with normal fasting glucose concentrations < 7.0mmol/l [22]. Insulin resistance was diagnosed using the Homeostatic Model Assessment for Insulin Resistance (HOMA-IR) values ≥ 3.42 based on previously used adolescent population-based reference ranges [23]. Due to the retrospective nature of this study, measurements were not done systematically but rather as guided by clinical suspicion (e.g. signs of acanthosis nigricans or weight gain).

### Statistical analysis

Non-parametric data was summarised with medians and ranges. The Mann-Whitney U test was used to assess for differences between groups with a p-value of < 0.05 being considered significant. Statistical analysis was performed using Microsoft Excel (Microsoft, Redmond, WA, USA) and RStudio (RStudio Team, Boston, MA).

### Ethics

This study was registered as a service evaluation project by the Joint Research and Development Office for Great Ormond Street Hospital (Study ID: 3836) and UCL Great Ormond Street Institute of Child Health. No ethics approval was therefore required.

## Results

### Patient characteristics

56 patients were identified from a search of electronic patient records, with 55 included in the final analysis. One patient was excluded from analysis as they had started treatment in another centre. Baseline characteristics of the cohort are summarised in Table 1. Median age at the point of data collection was 9.6 years (1.6–20.6) with the most common indication for treatment being high- and low-grade gliomas (including pilocytic astrocytomas). Other common indications for treatment included LCH and plexiform neurofibromas. One patient was treated for both a low-grade optic pathway glioma and a plexiform neurofibroma in the context of neurofibromatosis type 1. The remaining patients were treated for a variety of other indications (Table 1.). The most common coexisting endocrine comorbidities were growth hormone deficiency (*n* = 10, 18.2%) followed by precocious puberty (*n* = 9, 16.4%) and ACTH/adrenal insufficiency (*n* = 9, 16.4%).

**Table 1:** Demographic and treatment related characteristics. GH = growth hormone, ACTH = adrenocorticotropic hormone, TSH = thyroid-stimulating hormone, FSH = follicle-stimulating hormone, LH = luteinising hormone

	*n*/median (%/range)
Age	9.6 (1.6–20.6)
Sex	
Male	28 (50.9)
Female	27 (49.1)
Indication for treatment	
Glioma	34 (61.8)
Low-grade glioma	30 (54.5)
High-grade glioma	4 (7.3)
Langerhans cell histiocytosis	10 (18.2)
Plexiform neurofibromas	6 (10.9)
Craniopharyngioma	1 (1.8)
Central conducting lymphatic anomaly (Noonan syndrome)	1 (1.8)
Meningioma	1 (1.8)
Ganglioma	1 (1.8)
Ganglioneuroma	1 (1.8)
Congenital melanocytic nevi	1 (1.8)
Treatment	
Trametinib monotherapy	29 (52.7)
Dabrafenib monotherapy	12 (21.8)
Trametinib + dabrafenib	14 (25.5)
Endocrine Comorbidities	
GH^a^ deficiency	10 (18.2)
Precocious puberty	9 (16.4)
ACTH deficiency^b^	9 (16.4)
TSH^c^ deficiency	7 (12.7)
Diabetes insipidus	7 (12.7)
FSH^d^, LH^e^ deficiency	2 (3.6)

### Hyponatraemia

Prior to treatment, eight patients had at least one episode of hyponatraemia. However, two of these readings were more than 5 years prior to starting treatment. Four of these patients had a diagnosis of central diabetes insipidus.

During treatment nine patients had at least one episode of hyponatraemia, seven of whom were on trametinib (four as monotherapy). Their characteristics are summarised in Table 2. The median plasma sodium nadir of these patients was 133 (127–134) mmol/L. Three of these patients had a pre-existing diagnosis of central diabetes insipidus, with two having had an episode of hyponatraemia prior to starting treatment. Only one patient had an episode of hyponatraemia prior to treatment in the absence of central diabetes insipidus. A statistically significant difference (p-value = 0.037) with regards to the plasma sodium nadir during treatment (with either dabrafenib/trametinib or both) was observed between patients with diabetes insipidus (median = 134 (132–137) mmol/l) and patients without (median = 137 (127–141) mmol/l). Patients with diabetes insipidus had either low-grade gliomas, craniopharyngioma or LCH. For whom measurements were available, one patient who had ceased trametinib monotherapy had no post-treatment hyponatraemic readings (Table 2).

**Table 2:** Demographic and treatment related characteristics of patients diagnosed with hyponatraemia during treatment with dabrafenib or trametinib. ACTH = Adrenocorticotropic hormone, FSH = Follicle-stimulating hormone, GH = Growth Hormone, LH = Luteinising hormone, TSH = Thyroid-stimulating hormone

Sex	Diagnosis	Treatment	Endocrine Comorbidities	Pre-treatment hyponatraemia	Treatment sodium nadir (mmol/L)	Post-treatment hyponatraemia (0/1)
Male	Langerhans cell histiocytosis with pituitary involvement	Dabrafenib, trametinib	GH, LH, FSH, ACTH, TSH deficiencies central diabetes insipidus	No	134	N/A
Male	Suprasellar low-grade glioma	Dabrafenib	GH deficiency,Central precocious puberty	No	134	N/A
Male	Langerhans cell histiocytosis with pituitary involvement	Dabrafenib, trametinib	Central diabetes insipidus	Yes	132	N/A
Female	Low-grade optic pathway glioma, Plexiform neurofibroma	Trametinib	N/A	No	133	N/A
Female	Suprasellar low-grade glioma	Trametinib	ACTH, TSH deficiencies, central diabetes insipidus	Yes	133	N/A
Male	Suprasellar low-grade glioma	Dabrafenib	GH, TSH deficiencies	No	132	No
Female	Suprasellar malignant ganglioglioma	Dabrafenib, trametinib	N/A	No	134	N/A
Male	Langerhans cell histiocytosis of lung	Trametinib	N/A	No	132	No
Female	Cervicothoracic ganglioneuroma	Trametinib	N/A	Yes	127	Yes

### Glucose control dysfunction

Six patients were identified as having some form of glucose control dysfunction; three patients were diagnosed as having insulin resistance while another three were diagnosed as having impaired glucose tolerance (Table 3). Two patients were diagnosed prior to starting treatment (craniopharyngioma and low grade optic pathway glioma). The four patients diagnosed during treatment were all on dabrafenib, while the two diagnosed prior to treatment were on trametinib monotherapy. All four patients diagnosed during treatment also possessed suprasellar lesions (pilocytic astrocytomas, LCH and suprasellar malignant ganglioglioma). The median BMI Z score in this sub-cohort diagnosed with glucose control dysfunction during treatment was 1.37 and 2.38 prior to and during treatment respectively (p-value = 0.1). However in patients treated with dabrafenib not experiencing glucose control dysfunction a decrease in BMI Z-score was noted from 0.95, prior to treatment, to 0.73 at their latest reading during/post-treatment (median difference = 0.22, p-value = 0.9).

**Table 3:** Demographic and treatment related characteristics of patients diagnosed with impaired glucose tolerance/insulin resistance during treatment with dabrafenib or trametinib. ACTH = adrenocorticotropic hormone, FSH = follicle-stimulating hormone, GH = growth hormone, IGT = impaired glucose tolerance, IR = insulin resistance, LH = luteinising hormone, TSH = thyroid-stimulating hormone

Sex	Diagnosis	Endocrine Comorbidities	Treatment	Pre-treatment weight (Kg)	Pre-treatment BMI (Z-Score)	Treatment weight (Kg)	Post-treatment BMI (Z-Score)	BMI Z-score difference	Glucose control dysfunction
Male	Multisystemic Langerhans-cell histiocytosis	LH, FSH, ACTH, TSH deficiencies, central diabetes insipidus	Dabrafenib, trametinib	32.5	-0.2	64.7	1.0	1.2	IR
Female	Low-grade optic pathway glioma	GH, LH, FSH, ACTH, TSH deficiencies	Trametinib	66.2	2.8	62.5	N/A	N/A	IR
Female	Suprasellar low-grade glioma	GH, FSH, ACTH, TSH deficiencies	Dabrafenib, trametinib	24.0	2.3	55.5	2.8	0.6	IR
Female	Craniopharyngioma	GH, ACTH, TSH deficiencies central diabetes insipidus	Trametinib	73.0	2.9	73.5	2.9	-0.1	IGT
Male	Suprasellar low-grade glioma	GH, TSH deficiencies	Dabrafenib	49.7	1.3	89.8	2.8	1.4	IGT
Female	Suprasellar malignant ganglioglioma	N/A	Dabrafenib, trametinib	31.9	1.4	49.6	2.0	0.6	IGT

## Discussion

Our study demonstrates that despite their benefits, molecular therapies still bring their own unique off-target effects. In particular, our study suggests that dabrafenib and trametinib possess hyperglycaemic and hyponatraemic effects in paediatric patients respectively. The hyponatraemic effects of trametinib appear to be more pronounced in patients with co-existing diabetes insipidus, whilst the hyperglycaemic effects of dabrafenib appear to be more pronounced in patients with suprasellar lesions.

The hyponatraemic effect in patients with low-grade gliomas and coexisting diabetes insipidus has previously been reported in the literature [20]. Our study demonstrates that patients with co-existing diabetes insipidus appear more vulnerable to episodes of hyponatraemia. Although the reason for this predisposition is currently unknown, MEK inhibition is thought to possibly upregulate the insertion of aquaporins (AQP) in certain cells [24]. AQP1 in retinal pigment epithelium cells has been shown to be downregulated by MEK/ERK activation [25]. The MAPK pathway has previously been demonstrated to play a role in the insertion of AQP in renal medullary cells [26, 27]. Desmopressin, the mainstay of treatment for central diabetes insipidus, has been shown to decrease phosphorylation of ERK1/2, downstream of MEK, in collecting duct cells [28]. It could therefore be possible that MEK inhibition potentiates the antidiuretic effect of desmopressin. Some patients on trametinib have had doses of desmopressin reduced or withheld [20, 29]. Patients being treated with trametinib should therefore have regular review of serum sodium and desmopressin doses to ensure early detection and treatment.

The exact mechanism for dabrafenib induced hyperglycaemia has not been elucidated, given that glucose control mediated by insulin is primarily through the PI3K pathway rather than MAPK/ERK [30]. However, studies have shown Vemurafenib (BRAF inhibitor) alters uptake of Fludeoxyglucose (FDG) in melanoma cells harbouring the BRAFV600E mutation [31, 32], with changes being noted particularly in Glucose transporters 1/3 (GLUT1/3) [33]. Though such effects are useful in the treatment of cancers which thrive in glucose-rich environments, it could be possible these effects may occur in normal cells as well. Inhibition of the MAPK/ERK pathway through BRAF inhibitors may also result in disruption of the melanocortin-4 receptor pathway (MC4R). In humans, MC4R deficiency represents the commonest known monogenic obesity disorder [34]. The MAPK/ERK pathway has previously been implicated in MC4R signalling with MEK inhibition preventing the potentiation of MC4R on leptin induced STAT3 signalling and downstream anorexigenic effects [35]. The presence of suprasellar lesions in the patients diagnosed with glucose control dysfunction may predispose them to hypothalamic damage resulting in hyperinsulinaemia and hypothalamic obesity [36, 37]. The latter has previously been observed in patients with LCH and pilocytic astrocytoma [37, 38]. As the patients on dabrafenib that were diagnosed with glucose control dysfunction all had suprasellar lesions, it is unclear if their diagnosis was due to their treatment or to their underlying predisposition to endocrine dysfunction.

This is the largest cohort reported so far on the effects of MEK and BRAF inhibitors on sodium and glucose homeostasis for a range of indications being represented, thus allowing the frequency of off-target effects of molecular therapies to be estimated in populations other than low-grade gliomas. However, despite the larger sample size, this was still too small to allow us to perform more complex statistics to identify relevant associations and risk factors, such as the presence of endocrinopathies or tumour location, for the discussed side-effects. Furthermore, due to the retrospective nature of the study, a great deal of data was missing for the data we intended to collect. In particular, data on glucose control measurements was not regularly collected prior to the start of treatment. The number of patients who stopped treatment was small therefore preventing us from assessing the reversibility of these effects.

Ultimately, further prospective studies are needed to determine the endocrine morbidity that can be attributed to molecular therapies and whether these effects are reversible, especially with forms of glucose control dysfunction. Data collection on dysregulation of sodium and glucose homeostasis should be included in ongoing and future clinical trials of these medications. Additionally, clinicians using such treatments need to be aware of these potential effects, particularly the risk of hyponatraemia in patients with pre-existing central diabetes insipidus and monitor for these accordingly, including performing measurements of sodium and glucose prior to, during and after treatment.

## Data Availability

The datasets generated during and/or analysed during the current study are available from the corresponding author on reasonable request.
